# Editorial: The Relationship Between Neural Circuitry and Biomechanical Action

**DOI:** 10.3389/fnhum.2022.838028

**Published:** 2022-02-03

**Authors:** Redha Taiar, Mario Bernardo-Filho, Borja Sañudo, Yury Ivanenko

**Affiliations:** ^1^MATIM, Université de Reims Champagne-Ardenne Reims, Reims, France; ^2^Laboratório de Vibrações Mecânicas e Práticas Integrativas (LAVIMPI), Instituto de Biologia Roberto Alcântara Gomes and Policlínica Piquet Carneiro, Universidade do Estado do Rio de Janeiro, Rio de Janeiro, Brazil; ^3^Department of Physical Education and Sport, University of Seville, Seville, Spain; ^4^Laboratory of Neuromotor Physiology, IRCCS Fondazione Santa Lucia, Rome, Italy

**Keywords:** neuromechanics and control of physical behavior, musculoskeletal and neuromuscular biomechanics, rehabilitation in neuro-biomechanics, neuromechanical analysis in sport medicine, neuromuscular training

In this Research Topic the relationship between neural circuity and biomechanical action of the musculoskeletal system is addressed. Biomechanics studies the complexity of the human behavior including dynamic motion models (i.e., balance and gait), consequently biomechanics and neural control of movement are interrelated. The control of different body segments is required by neurological and biomechanical management of human movement that can be coordinated in multiple ways to perform a defined task. To control a movement of a target in a task, the individual can use an infinite number of segmented trajectories that remain under the control of several actuators (muscles). This is all based on different types of sensory information sources that the individual is receiving. Comprehending how an individual's central nervous system directs all different levels of redundancy and how it manages to propose an optimal solution to perform the procedure remains a scientific challenge. Various studies have been developed to add information in better understanding of all this complex relationship between the coordination of neural circuitry and biomechanical action in the musculoskeletal system. Coordination is one of the central concepts in human movement science, especially in the field of biomechanics and motor control. Biomechanics is a field that aims to identify the function of elements (e.g., muscles, joint movements) involved in achieving motor tasks. This identification is expected to provide information on improving movement and making it safer. Nevertheless, because the musculoskeletal system is highly interconnected and integrated, it is almost impossible to identify the function of each element in isolation during a whole-body movement.

The aim of this Research Topic was to summarize the most important neuro-biomechanical parameters influencing human performance related to the health sciences and sports in individuals with different ages and with various clinical conditions. Besides the information acquired from clinical interventions or experimental models, mathematical point of view, understanding this interaction means solving systems of under-determined equations (i.e., systems that have more unknowns than equations), and therefore an infinity of solutions are possible. Moreover, the use of several tools is also relevant to access information related to the mechanism involved with neural circuits and responses associated with movements.

The collection of articles in this Research Topic covers a range of issues such as conceptual frameworks, neuromechanical assessment and analysis, neuromodulation designs, coordination, and biomechanics relating to training. Studies involving populations with Spinal Cord Injury individuals, Parkinson's Disease, Stroke, Autism Spectrum Disorder, infants and healthy adults are presented and different strategies were used to access biological responses. This Research Topic includes 26 contributions (3 reviews, 1 mini review, 2 systematic reviews, 1 perspective, 1 opinion paper, 17 original articles, and 1 case report) that are summarized below in 4 thematic categories: (i) reviews and perspectives, (ii) neuromechanical assessments, (iii) coordination assessments, and (iv) biomechanics related to neuromuscular training and neuroplasticity in rehabilitation and sport.

## Reviews and Perspectives

Several articles present and discuss available evidence, conceptual frameworks, and fundamental questions concerning the relationship between neural circuits and biomechanical action from different perspectives, including motor development, basic control mechanisms and clinical applications.

Dewolf et al. discuss the relationship between neural circuitries and changing biomechanics from the developmental point of view. The authors review neural and biomechanical mechanisms underlying walking and running gaits, their development in early childhood, biomechanical determinants, differentiating features, and the neuromechanical underpinnings of early gait maturation. They also consider the neuromuscular maturation time frame of gaits resulting from active practice and the underlying plasticity of development. Bridging connections between movement mechanics and neural control of movement could have profound clinical implications for technological solutions to better understand locomotor development and to diagnose early motor deficits. In particular, in a perspective article, Gygax et al. present converging recent knowledge in neuroscience and biomechanics to outline the relationships between maturing neuronal network, behavior, and neurodevelopmental disorders. The authors argue that developmental gait biomechanics might appear as a possible motor phenotype and biomarker to be correlated to neuronal network maturation, in normal and atypical developmental trajectories—-such as autism spectrum disorder.

In the context of the interrelationship between biomechanics and neural control of movement, Kimura et al. consider coordination as a multidisciplinary concept for accurate interpretation of data and theory development. By comprehensively providing multiple perspectives on coordination from computational and ecological perspectives, and the meaning of this term, this review article intends to promote coordination studies in biomechanics and neural control. In the context of neuromechanical functioning of different body segments, Geroza and Lombardi review the multiple roles that emerged for bone tissue as the principal mechanosensitive organ. Particularly, its high innervation, sensitivity to mechanical stimuli, the endocrine function, the capability to sense and integrate different stimuli and to send signals to other tissues, allow the adaptation of the affected bony segment to the changing environment and its communication with the CNS. As exercise effectively modifies the release of osteokines, it has been hypothesized that some of the beneficial effects of physical activities on brain functions may be associated with such a bone-to-brain communication for the treatment of neurodegenerative diseases.

Several articles discuss the use of neuromodulation tools to access biomechanical responses, the underlying neural mechanisms, and related clinical interventions. In a mini review article, Wang and Choi review the physiological and pathophysiological roles of basal ganglia and cortical oscillations, as well as their interactions, in specific biomechanical manifestations of pathological gait. In particular, the authors consider the functioning of the brain network for the control of gait in Parkinson's disease (PD), where specific patterns of abnormal oscillatory synchronization in the basal ganglia thalamocortical network are associated with specific signs and symptoms. They also discuss potential therapies aimed at restoring gait impairments through modulation of the brain network in PD. Two systematic reviews included in the Research Topic assess the current knowledge of the effect of transcranial stimulation. Xiao et al. review the literature on the effect of transcranial direct current stimulation (tDCS) on the physical performance of the foot and ankle of healthy adults and discuss the underlying neurophysiological mechanisms through which cortical activities influence the neuromechanical management. tDCS may induce remarkable improvements in the physical performance, including vibratory and tactile threshold of the foot sole, toe pinch force, ankle choice reaction time, accuracy index of ankle tracking, and ankle range of motion, compared to sham. Fan et al. review the literature on the effect of repetitive transcranial magnetic stimulation (rTMS) in inducing neuroplastic changes and promoting brain function restoration in stroke patients for the treatment of lower-limb motor dysfunction and improving the neuro-biomechanical parameters of gait. Finally, in an opinion article, Moshonkina et al. review the literature and discuss a promising approach of non-invasive strategy for spinal neuromodulation (scTS) to control human locomotion that might be effective for neuromuscular control of postural and locomotor function in post-stroke subjects and in individuals with spinal cord injury (SCI). The authors introduce a strategy of spinal neuromodulation using the continuous stimulation to activate the locomotor networks in combination with rhythmic targeted activation of flexor and extensor motor pools of leg muscles in different phases of step cycle in accordance with the biomechanical function of these muscles.

## Neuromechanical Assessments

Neuromechanical assessments represent a powerful tool to investigate the relationships between neural circuitry and biomechanical action. Grooms et al. report a kinetically-instrumented neuroimaging evaluation of neural correlates of force control using fMRI. The authors document that knee extension and flexion force-matching tasks increase BOLD signal among cerebellar, sensorimotor, and visual-processing regions, and reveal some unique activation strategies depending on whether engaging knee extension or flexion. They argue that a neuroimaging-compatible force control paradigm may serve as a method to investigate how pathologies affect lower extremity neuromuscular function. Martino et al. examine associations between biomechanical outcomes and clinical leg rigidity score in people with Parkinson's disease (PD) by recording kinematic data and electromyographic signals during the pendulum test. The results suggest that the biomechanical assessment of the pendulum test can objectively quantify parkinsonian leg rigidity and be associated with a history of falls. Different mechanisms that contribute to resting and activated rigidity can play an important functional role in balance impairments and be used for neuromechanical assessments.

Several studies consider the anatomical, functional and behavioral factors for neuromechanical assessments. Nobue et al. examine the relationship between nerve conduction velocity (NCV) and nerve size in healthy subjects using supramaximal electric stimulation and peripheral nerve ultrasonography. The results show that the NCV, nerve cross-sectional area (nCSA), and circumference of the ulnar and tibial nerves were higher and greater in the lower limbs than in the upper limbs. The results also suggest that NCV does not depend on the nCSA sizes or upper and lower limb circumference and thus they indicate the existence of limb-specific NCV but not nCSA developments. Knothe Tate et al. address the need to image and analyse cellular connectivity across length and time scales through development of technological approaches that incorporate cross length scale (nm to m) structural data, acquired *via* multi-beam scanning electron microscopy, with machine learning and information transfer using network modeling approaches. Cells constitute biological materials of living organisms that exhibit “smart” stimuli-responsive and adaptive behavior, including changes in cellular connectivity and tissue remodeling by cells. The authors discuss the implications of the outlined approach for neuromechanics and the control of physical behavior and neuromuscular training, and for understanding the cellular underpinnings of diseases. Weinman et al. report the influence of various behavioral factors on long-latency (involving cortical areas) responses to perturbations. They examine the effect and interaction of such factors as background muscle torque, perturbation direction, perturbation velocity and task instruction, and argue about feedback processes in the CNS to control interactions with the environment. Recenti et al. use a novel multimetric system to study the physiology associated with motion sickness and sickness and to evaluate the associated postural control disturbances. The multimetric system was based on using a mechanical moving platform, virtual reality, EMG, EEG and heart rate recordings. The feature importance analysis showed that muscle parameters are the most relevant, and for EEG analysis, beta wave results were the most important. The present work serves as the first step in identifying the key neurophysiological factors that differentiate those who suffer from motion sickness from those who do not.

The role of biomechanical factors is also described in assessing grasping, speech and postural control. Nataraj and Sanford describe how modifications in the display of a computer trace can co-modulate agency (perception of control) and performance of grasp on rigid and compliant surfaces. Agency and performance of grasp can be co-modulated across varying modes of control, especially for compliant grasp actions. The implications of this work are cognition-centered device interfaces for the rehabilitation of grasp after neurotraumas while considering whether the grasp interaction is rigid or compliant. Gómez et al. assess performance, neuromotor (EMG) activity, and distributions of the kinematic and acoustic velocities extracted from the speech signal during the utterance of a diadochokinetic exercise to characterize Hypokinetic Dysarthria in participants with PD. The regression results show the relationships between EMG and dynamic and acoustic estimates, significant cross-correlations between articulation kinematics, and suggest that kinematic distributions derived from acoustic analysis may be useful biomarkers toward characterizing Hypokinetic Dysarthria in neuromotor disorders. Lhomond et al. examine whether the brain uses an internal model of physical laws (gravitational and inertial forces) to help estimate body equilibrium when tactile inputs from the foot sole are depressed by carrying extra weight. To this end they analyzed EEG characteristics and neural responses to tactile stimulation of the foot sole and compared them in Judoka athletes with non-athlete participants and dancers. They show smaller amplitudes of P1N1 somatosensory cortical potential in the Load compared to the No Load condition in both non-athletes and dancers. This decrease in neural response to tactile stimulation was associated with greater postural oscillations. Along with improved postural reactions evoked by a translation of the support surface in Judokas, the results suggest that an internal model stored in the right PPC can optimize predictive mechanisms in situations with high balance constraints.

## Coordination Assessments

Even the simplest movement engages many brain structures and necessitates not only activation but also inhibition of relevant neural circuits since the same motoneurons and interneurons participate in a huge repertoire of possible motor actions. For the complex movements, coordination is an important concept with which the CNS must deal when controlling multiple muscles and multiple degrees of freedom. A number of articles in this Research Topic explore coordination assessments to get insights into the relationship between neural control and biomechanics of movement involving multiple body segments.

Four articles address the issue of coordination patterns of human gait. Stetter et al. assess modularity in motor control by analyzing kinematic synergies across varying locomotor tasks: straight-line walking, walking a 90° spin turn, and walking upstairs. Covariation of joint motions can be analyzed and interpreted in the context of kinematic synergies relating to the control mechanisms underlying human motor behavior. The findings further support the idea that movements can be performed efficiently through a flexible combination of a lower number of control-relevant variables. Bach et al. assess multi-muscle coordination in children of different age (2–9 yrs) by evaluating muscle synergies that were proposed to reflect the presence of a common neural input to multiple muscles. By analyzing the activation patterns of 15 bilateral leg, trunk, and arm muscles, ground reaction forces, and kinematics of walking and running, including the “walk-run strategy” in pre-schoolers, the authors investigated the development of different gait control. In particular, the findings suggest that the increase in the number of muscle synergies in older children can be related to motor learning and exploration. Krajewski et al. examine the complexity and stability of the state of the locomotor control system by analyzing motor variability using a goal equivalent manifold approach. They assess the interactive effects of load magnitude and locomotion pattern on motor variability, stride regulation and gait complexity during 1-min trials of running and forced marching in healthy young adult women. Reduction in “good” variability as load increases and load-related decrease of gait complexity are interpreted as important characteristics and changes in the locomotor system function. Unni et al. investigate disruptions in the coordination pattern during an involuntary stopping of gait in late-stage PD patients, known as freezing of gait. They use a neuromechanical model of gait to infer the causes of both the observed variability and freezing in PD. The model reveals that the opposing forces generated by the plantar flexors of the swing and stance leg can induce freezing, as well as other gait abnormalities near freezing such as a reduction in step length, and irregular walking patterns. The study by Maeda et al. explores the eye-head coordination mechanisms by analyzing the effects of high frequency noisy vestibular electrical stimulation delivered by electrodes placed on mastoid processes. The coordination of eye–head movements was measured by eye-tracking and a motion capture system. The authors report that this stimulation can reduce the lag time between eye and head movement and improve coordination, contributing to a clear retinal image and argue that this technique could be applied as a form of vestibulo-ocular reflex (VOR) training for patients with vestibular hypofunction.

## Biomechanics Related To Neuromuscular Training and Neuroplasticity In Rehabilitation and Sport

Several studies report and analyse the effects of training in rehabilitation and sport and the benefits of using neuromechanical outcomes and correlations for their assessment. The study by Simis et al. looked for a neurophysiological biomarker for functional recovery after robotic-assisted gait training in individuals with incomplete SCI. Functional impairments and improvements in balance and walking performance following rehabilitation therapy were correlated with the change in cortical activity measured by EEG. The results suggest that the EEG alpha/theta ratio may be a potential surrogate marker of functional improvement during rehabilitation. In a case report, Momeni et al. evaluate the biomechanical, neural, and functional outcomes of neuromuscular electrical stimulation (ES) in a supine position and combined with stand training using a body weight support system in an individual with spinal cord injury (SCI). The post test of ES alone showed gains in trunk independence with a decrease in lower limb muscle activation, while ES combined with stand training showed gains in trunk independence and increased muscle activation in trunk and lower limb muscles during the treadmill stepping paradigm. The results of the study illustrate the importance of loading during the stimulation for neural and mechanical gains. Huang et al. show the effectiveness of in-bed wearable elbow robot training in improving biomechanical and clinical outcomes in patients with early and late subacute stroke. Based on muscle strength recovery curve, the results also suggest that patients with severe upper limb motor impairment may benefit more from the robot training compared to those with moderate impairment. Purdom et al. report the effect of accumulated competition training stress on neuromuscular function and the incidence of increased injury risk in uninjured female athletes. They tested mobility/stability, leg length symmetry, and vertical power at three different points throughout the competitive season. The results show that competition stress affected neuromuscular function without affecting maximal power, which negatively affected stability.

Taken together, the articles compiled in this Research Topic demonstrate the growth of interest to using neuromechanical assessments for understanding the relationship between neural circuitry and biomechanical action. These works also show the importance of investigating the pathophysiology underlying these relationships to better develop assessments of impaired motor function and its restoration in patients with neurological disorders.

## Author Contributions

All authors listed have made a substantial, direct, and intellectual contribution to the work and approved it for publication.

## Funding

This work was supported by *Conselho Nacional de Desenvolvimento Cient*í*fico e Tecnológico (CNPq)* and *Fundação de Amparo á Pesquisa do Estado do Rio de Janeiro (FAPERJ)*, Brazil and the Italian Ministry of Health (Ricerca Corrente, IRCCS Fondazione Santa Lucia, Ricerca finalizzata RF-2019-12370232).

## Conflict of Interest

The authors declare that the research was conducted in the absence of any commercial or financial relationships that could be construed as a potential conflict of interest.

## Publisher's Note

All claims expressed in this article are solely those of the authors and do not necessarily represent those of their affiliated organizations, or those of the publisher, the editors and the reviewers. Any product that may be evaluated in this article, or claim that may be made by its manufacturer, is not guaranteed or endorsed by the publisher.

